# MINOCA mimic: Type 2 myocardial infarction due to severe aortic stenosis complicated by cardiogenic shock

**DOI:** 10.21542/gcsp.2023.1

**Published:** 2023-01-30

**Authors:** Momin Islam, Hussam Al Hennawi, Mohamad Bakir, Anwar Khedr, Sachin S. Goel

**Affiliations:** 1 Houston Methodist Hospital, Department of Cardiology, USA; 2 Department of Internal Medicine, Jefferson Abington Hospital, Abington, PA, USA; 3 Alfaisal University, College of Medicine, Riyadh, Saudi Arabia; 4 Mayo Clinic Health System, Mankato, Minnesota, USA

## Abstract

Acute myocardial infarction without significant obstructive coronary disease presents a challenging clinical entity that requires timely intervention. The term myocardial infarction with nonobstructive coronary arteries (MINOCA) describes a working diagnosis attributed to varying etiologies in patients with a presumed ischemic cardiac condition. Several overlapping etiologies can be classified as type 2 myocardial infarction (MI). The 2019 AHA statement established diagnostic criteria and clarified the associated confusion, aiding in appropriate diagnosis. In this report, we present a case of demand-ischemia MINOCA and cardiogenic shock in a patient with severe aortic stenosis (AS).

## Background

Although type 2 myocardial infarction (MI) has been recognized for years, MINOCA (myocardial infarction in the absence of obstructive coronary artery) is a new term. The presence of MI and lack of obstructive disease on coronary angiography are required for the diagnosis of MINOCA^1^. Approximately 90% of patients with MI have evident coronary artery obstruction (stenosis greater than 50%). The degree of stenosis in the remaining 10% of patients is less than 50%, and such patients are referred to as having (MINOCA)^2^. According to several large-scale registry investigations, the prevalence of MINOCA in the MI population ranges between 1 and 15%^3, 4^. Compared with individuals presenting with MI with obstructive CAD, patients with MINOCA show lower cardiac biomarker elevation and subtle electrocardiogram (ECG) findings^5^. Adults under the age of 55 years were more likely to be diagnosed with MINOCA. Those with MINOCA are more likely to be female and of Non-Anglo-Saxon ethnicity^3, 5^. MINOCA is a complex condition with multiple etiologies, including Takotsubo syndrome and coronary artery dissection. There are no angiographic findings of occlusion in the major epicardial vessels in patients with MINOCA; rather, myonecrosis is caused by distal embolization due to plaque rupture^1^. Compensatory expansion of stenotic epicardial vessels as a result of atherosclerosis is frequently a cause of angiographic underestimation, creating the impression of normal or minimally stenotic coronary arteries^6^. This pathophysiology is important to keep in mind since there is overlap with type 2 MI, which is defined as “ischemia without unstable coronary artery disease due to a mismatch in myocardial oxygen supply and demand”^1^.

## Case presentation

A 56-year-old man with a medical history of hypertension and hyperlipidemia was brought to the emergency department (ER) after an episode of syncope. He regained consciousness, but remained dizzy and called for an ambulance. The patient developed ventricular tachycardia on the way to the hospital, requiring brief CPR. He subsequently developed unstable atrial fibrillation with a rapid ventricular response (RVR) when he arrived at the ER, necessitating defibrillation.

**Figure 1.**
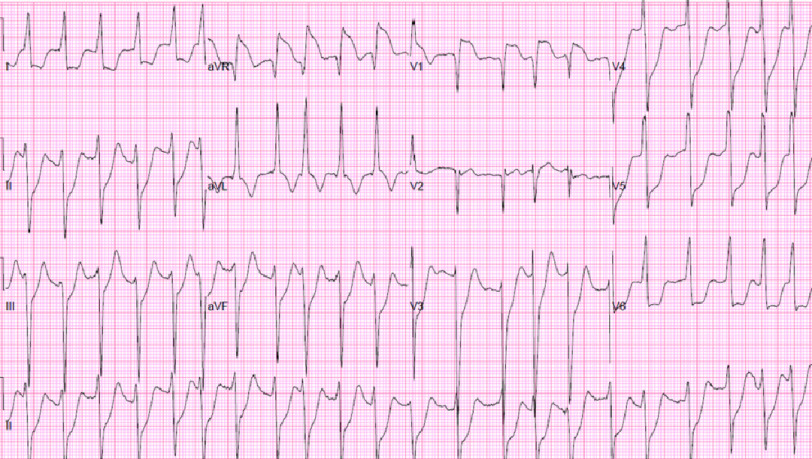
EKG showing ST elevation in AvR and V1 with global ST depression.

He also complained of chest pain, palpitations, dyspnea on exertion, bilateral lower-extremity edema, orthopnea, paroxysmal nocturnal dyspnea, nausea, and bloating. Physical examination revealed hemodynamic instability with a blood pressure of 76/48 mmHg, heart rate of 115 beats per minute, respiratory rate of 30 breaths per minute, and oxygen saturation of 95% on a 3 L nasal cannula. The patient appeared ill and diaphoretic. Chest examination revealed clear lung fields without wheezing or crackles. Lower limb pitting edema was evident bilaterally. The patient was defibrillated, and an electrocardiogram (EKG was performed, which revealed ST elevation in AvR and V1 with global ST depressions [Figure 1].

**Figure 2.**
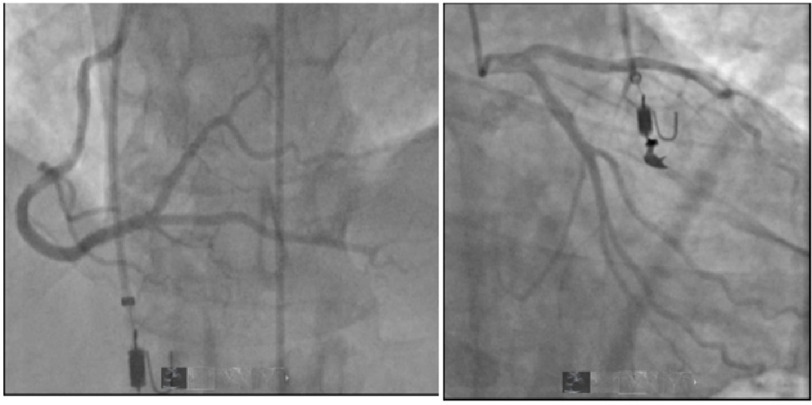
Heart catheterization revealed no evidence of obstructive coronary artery disease in the left main, left anterior descending artery (LAD), left circumflex coronary artery (LCx), or right coronary artery (RCA).

**Figure 3.**
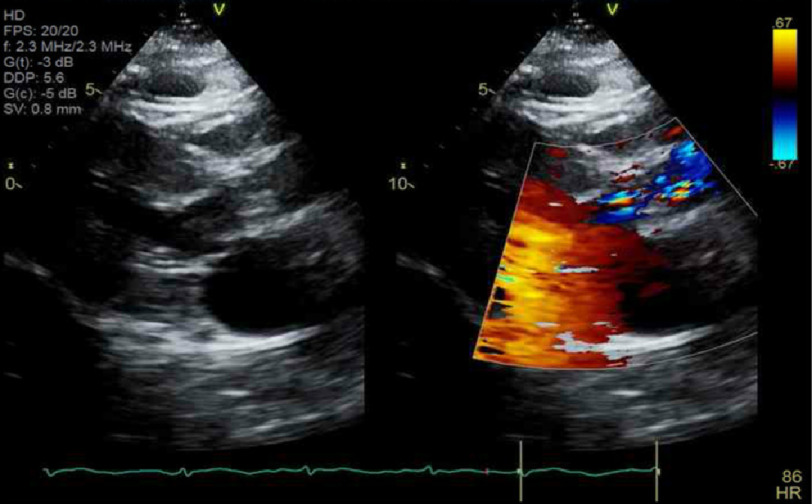
Transthoracic echocardiogram (TTE) showing a heavily calcified aortic valve with severe aortic valve stenosis and an aortic valve area of 0.25 cm^2^.

On laboratory investigation, the patient had an elevated troponin level of 130 ng/l (the normal range for men is less than 22 ng/L). A transthoracic echocardiogram (TTE) revealed an ejection fraction (EF) of 55–60%, severe left ventricular concentric hypertrophy, a heavily calcified aortic valve with severe aortic valve stenosis, peak aortic jet velocity of 4.4 m/s, a peak and mean gradient of 74 and 51 mmHg respectively and calculated aortic valve area of 0.25 cm^2^ [Figure 3]. Left heart catheterization (LHC) revealed no evidence of significant stenosis in any of the large epicardial arteries [Figure 2]. A transthoracic echocardiogram (TTE) revealed an ejection fraction (EF) of 55–60%, severe left ventricular concentric hypertrophy, a heavily calcified aortic valve with severe aortic valve stenosis, peak aortic jet velocity of 4.4 m/s, a peak and mean gradient of 74 and 51 mmHg respectively, and calculated aortic valve area of 0.2 5 cm^2^ [Figure 3].

During LHC, the patient began to desaturate and became hemodynamically unstable. He continued to require pressors for hypotension and atropine for bradycardia. He experienced respiratory arrest and required endotracheal intubation. Despite being on three pressors (epinephrine, norepinephrine, and phenylephrine), the mean arterial pressure (MAP) was 60 mmHg, cardiac index (CI) was 1.5 L/min/m^2^, central venous pressure (CVP) was 20 mmHg, and mixed venous oxygen saturation (SvO2) was 50%. The patient’s findings were consistent with cardiogenic shock, likely due to severe AS. Inotropic support with dobutamine and an intra-aortic balloon pump (IABP) was initiated to increase forward flow with the goal of achieving CI of 2.5–4 L/min/m^2^ and SvO2 of 60%, and diuresis was later initiated to assist in lowering CVP to 12 mmHg.

The patient was then transferred to our institution for further care. He was too unstable to undergo transcatheter aortic valve replacement (TAVR) or surgical aortic valve replacement (SAVR). After multidisciplinary heart team discussions, it was decided to proceed with balloon aortic valvuloplasty (BAV). He underwent BAV with a 20 mm, 22 mm, and 24 mm True balloon, which resulted in a reduction in the mean trans-aortic gradient from 48 mmHg to 13 mmHg. Epinephrine, norepinephrine, and phenylephrine were weaned off, followed by dobutamine over the next few days. The patient’s readings post balloon valvuloplasty and off-pressors were MAP >65 mmHg, CI 2.2 L/min/m^2^, SvO2 60%, and CVP 16 mmHg. Subsequently, however, the patient started to show clinical deterioration again, with hemodynamic instability, renal failure, respiratory failure, and encephalopathy without neurological improvement. After prolonged hospital stay and poor prognostication from a neurological standpoint, his wife decided to withdraw care and he died in the hospital.

## Discussion

Myocardial infarction with nonobstructive coronary arteries is a challenging clinical entity, present in up to 6% of patients diagnosed with acute myocardial infarction^7^. The 2019 AHA consensus sheds light on some unclear objectives specific to the diagnostic term MINOCA. To establish the diagnosis, either an increase or decrease in cardiac troponin (defined as a 20% change) with one value >99th percentile, confirmed evidence of infarction, which may be explained by symptoms consistent with myocardial ischemia, absence of obstructive coronary artery disease on angiography (no stenosis ≥50% in any major epicardial vessel), and exclusion of any alternate diagnosis explaining the clinical presentation. While this case was initially thought to be MINOCA after LHC lacking significant CAD, the ischemia was explained by an alternative diagnosis of critical aortic stenosis and thus classified as Type 2 MI.

Severe aortic stenosis may engender a myriad of cardiac changes, including structural and autoregulatory impeding coronary flow reserve and myocardial ischemia in the absence of coronary artery disease. Structurally, left ventricular hypertrophy as result of significant aortic stenosis, increases myocardial oxygen demand and interferes with coronary circulation through direct extravascular compression limiting myocardial perfusion. The course of MINOCA is dependent on its etiology. Such causes can be categorized as plaque disruption, coronary artery vasospasm, microvascular dysfunction, coronary embolism, spontaneous coronary artery dissection, and supply demand mismatch, as in our patient^8^.

According to the Variation in Recovery: Role of Gender on Outcomes of Young Acute MI Patients (VIRGO) trial, determining the cause of MINOCA influences patient prognosis and, more importantly, management^9^. Cases caused by plaque disruption, coronary embolism, and coronary artery dissection can benefit from aspirin, statins, and angiotensin-converting enzyme inhibitors (ACEi)^8^. Coronary artery vasospasm and microvascular dysfunction are better addressed with anti-anginal therapies in addition to previous medications^8^. The supply–demand mismatch is treated by addressing the underlying cause.

In this case report, our patient had severe type 2 MI due to supply–demand mismatch and severe AS which showed clinical improvement after balloon valvuloplasty. The Swedish Coronary Angiography and Angioplasty Registry (SWEDEHEART) trial showed that patients with MINOCA after a mean follow-up of 4 years had a significantly lower all-cause mortality rate, hospitalization for MI, ischemic stroke, and heart failure after taking aspirin, statins, and ACEi^10^. The MINOCA BAT study is ongoing and plans to stratify 3500 patients with MINOCA to treatment with ACEIs/ARBs and *β*-blockers or matching placebo to determine whether these therapies provide protection against major adverse cardiac events^11^. By being more cognizant of MINOCA and its various etiologies, physicians can offer treatments better tailored to their patients’ needs, thus improving cardiac morbidity and mortality^12^.

## Conclusion

In this case report, we present a case of Type 2 MI demand-ischemia complicated by cardiogenic shock in critical AS. This case is not consistent with MINOCA due to the valvular etiology. The supply–demand mismatch was addressed by treating the underlying cause. Our patient was unable to get TAVR or SAVR for AS due to his hemodynamic instability and continued to deteriorate despite BAV. For other cases of MINOCA and type 2 MI, medical management can be tailored for different etiologies.
